# Diets, nutrients, genes and the microbiome: recent advances in personalised nutrition

**DOI:** 10.1017/S0007114521000374

**Published:** 2021-11-28

**Authors:** Nathan V. Matusheski, Aoife Caffrey, Lars Christensen, Simon Mezgec, Shelini Surendran, Mads F. Hjorth, Helene McNulty, Kristina Pentieva, Henrik M. Roager, Barbara Koroušić Seljak, Karani Santhanakrishnan Vimaleswaran, Marcus Remmers, Szabolcs Péter

**Affiliations:** 1 Nutrition Science and Advocacy, DSM Nutritional Products LLC, Parsippany, NJ, USA; 2 Nutrition Innovation Centre for Food and Health (NICHE), School of Biomedical Sciences, Ulster University, Coleraine BT52 1SA, Northern Republic of Ireland; 3 Department of Nutrition, Exercise and Sports, Faculty of Science, University of Copenhagen, Rolighedsvej 26, 1958 Frederiksberg, Frederiksberg, Denmark; 4 Jožef Stefan International Postgraduate School, Jamova cesta 39, 1000 Ljubljana, Slovenia; 5 Hugh Sinclair Unit of Human Nutrition, Department of Food and Nutritional Sciences, University of Reading, Whiteknights, Reading RG6 6DZ, UK; 6 Computer Systems Department, Jožef Stefan Institute, Jamova cesta 39, 1000 Ljubljana, Ljubljana, Slovenia; 7 Koninklijke DSM N.V., Geleen, The Netherlands; 8 Nutrition Innovation Center, DSM Nutritional Products Ltd, Kaiseraugst, Switzerland

**Keywords:** Personalised nutrition, Epigenetics, Folate, Vitamin B_12_, Obesity, Microbiome, Deep learning

## Abstract

As individuals seek increasingly individualised nutrition and lifestyle guidance, numerous apps and nutrition programmes have emerged. However, complex individual variations in dietary behaviours, genotypes, gene expression and composition of the microbiome are increasingly recognised. Advances in digital tools and artificial intelligence can help individuals more easily track nutrient intakes and identify nutritional gaps. However, the influence of these nutrients on health outcomes can vary widely among individuals depending upon life stage, genetics and microbial composition. For example, folate may elicit favourable epigenetic effects on brain development during a critical developmental time window of pregnancy. Genes affecting vitamin B_12_ metabolism may lead to cardiometabolic traits that play an essential role in the context of obesity. Finally, an individual’s gut microbial composition can determine their response to dietary fibre interventions during weight loss. These recent advances in understanding can lead to a more complete and integrated approach to promoting optimal health through personalised nutrition, in clinical practice settings and for individuals in their daily lives. The purpose of this review is to summarise presentations made during the DSM Science and Technology Award Symposium at the 13th European Nutrition Conference, which focused on personalised nutrition and novel technologies for health in the modern world.

## Graphical abstract

**Figure:**
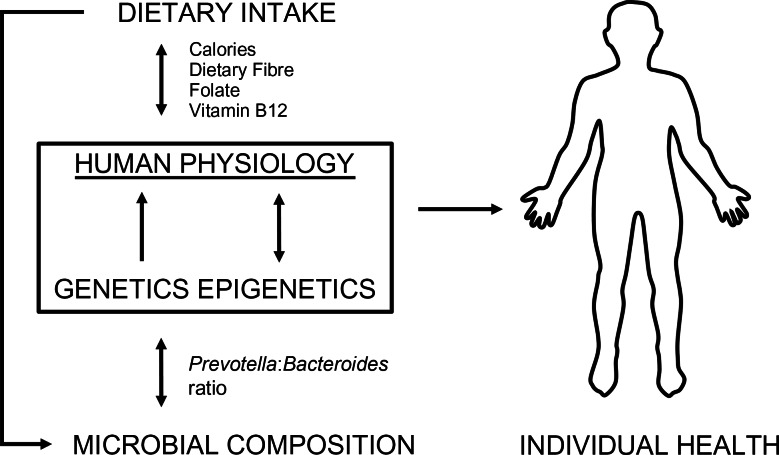

Although most dietary recommendations today are based on population averages, it is now possible to identify population sub-groups, and even individuals, who may benefit from modified nutrition guidance. As technology continues to advance, it is becoming easier for individuals to determine when they may be at increased risk for disease or a nutritional deficiency. They can access commercially available tools that provide precise guidance relevant to their genetics, phenotype and preferences^(1)^. However, personalised nutrition approaches, such as consumer nutrigenetics testing, have come under scrutiny^(2)^. Such systems require robust scientific substantiation, as recently described in a set of guiding principles^(3)^ and outlined by the Academy of Nutrition and Dietetics for application to dietetic practice^(4)^. Over the past several decades, it has become increasingly clear that a complex set of interactions exist among the genetic and environmental variables affecting individual responses to diet and lifestyle behaviours. Since the early 1990s, candidate gene studies have identified several genetic variations which have led to the understanding of the pathophysiological mechanisms and pathways that underlie complex diseases^(5)^. In the last 15 years, significant advances have been made through genome-wide association studies, with the discovery of several novel loci for complex traits^(6)^. However, these studies have shown that only a small amount of individual variability can be explained by genetics alone^(7)^. More recently, a holistic view has emerged regarding the interaction of genetics and other individual factors^(8)^. Such interactions can be behavioural, like the relationships between individuals and their food preferences and dietary intake, or physiological, such as genotype or microbiome. These interactions, and the underlying individual variations that drive them, play a significant role in personal health and well-being over time. However, they also challenge our existing approaches to research and complicate the application of research findings for the development of personalised nutrition guidance.

In this paper, we review four recent advances in the field of personalised nutrition that can lead to an improved understanding of individual health: (1) the influence of epigenetic changes and gene expression on cognitive function in early life, (2) the association genotype with nutrient status and corresponding metabolic responses in the context of obesity, (3) the relationship of gut microbial composition (enterotype) with the efficacy of fibre-rich dietary interventions for body weight management and (4) advanced technologies such as deep learning to improve the accuracy of dietary intake measurements. By examining these sources of inter-individual variability, and considering their possible interactions, we can better understand the nuances of individualised guidance and better define future research approaches to advance both personal and public health.

## Folate during pregnancy, epigenetic changes and child health outcomes

Maternal nutrition during pregnancy is essential for optimal offspring development, reducing lifelong disease burden and optimising health throughout life^(9)^. In particular, folate is required for one-carbon metabolism, a network of metabolic pathways involved in nucleotide synthesis, DNA repair and numerous methylation reactions^(10)^. In early pregnancy, there is conclusive evidence that periconceptional folic acid supplementation prevents neural tube defects^(11,12)^. Maternal folate supplementation may also affect neurocognitive development in early life^(13)^. This section focuses on the evidence linking folate nutrition in pregnancy, DNA methylation changes and health outcomes in the child.

### Folate, DNA methylation and brain development during pregnancy

Inadequate folate intake can interfere with early brain development and function, the consequences of which can vary depending on the timing of the deficiency relative to the development of the neurological structures^(14)^. The third trimester of pregnancy until 2 years after birth is a critical period of rapid growth and development of some regions of the brain such as cortical and subcortical grey matter^(15)^. Therefore, the continuation of folic acid supplementation beyond the first trimester (after the recommended period for the prevention of neural tube defects) may optimise folate status for prenatal brain development^(14,16)^. As such, maternal folate insufficiency appears to influence the developing brain^(17)^, which may result in lasting changes in child brain function^(13)^.

The effect of folate nutrition during pregnancy on health outcomes in the child is thought to involve the essential role of folate in one-carbon metabolism, which may impact neurodevelopment^(10,18)^. Folate-related epigenetic changes, and specifically DNA methylation, have been proposed as a plausible mechanism underpinning associations between maternal folate nutrition and various offspring health outcomes^(19)^. Epigenetic processes, including histone modifications, RNA interference and DNA methylation, involve changes to the genome that can alter gene expression without changing the underlying DNA sequence^(20)^. Early life development, ranging from preconception to childhood, is considered to be a critical window of the lifecycle, characterised by rapid DNA methylation changes, susceptibility to environmental factors and programming of epigenetic marks that may have lasting health effects in infants born to mothers with inadequate nutritional status^(21)^.

Observational studies have reported that folic acid supplement usage by women during pregnancy was associated with changes in DNA methylation patterns of candidate genes in cord blood^(22)^, with decreased methylation of *LINE-1* and *PEG3* and increased methylation of *IGF2* in cord blood^(23)^ and offspring^(24)^. However, associations between maternal folate exposure and the offspring methylome have been inconsistent^(25)^.

The Folic Acid Supplementation in the Second and Third Trimester (FASST) trial is thus far the only randomised trial of folic acid supplementation in pregnancy examining DNA methylation and maternal folate and homocysteine responses and related effects in the newborn^(26)^. In an analysis of biobanked samples from this trial, changes were found in DNA methylation of *LINE-1* and candidate genes related to brain development such as *IGF2* and *BDNF*, in the newborns of mothers who received folic acid^(27)^. Using an epigenome-wide (EWAS) approach, changes in newborn DNA methylation at the imprint regulator *ZFP57* were also shown^(28)^.

### Cognitive performance in offspring related to folate nutrition in pregnancy

Several observational studies have shown positive associations between self-reported folic acid supplement use in early pregnancy and childhood neurodevelopmental outcomes such as language delay, cognitive function score and verbal and motor function^(29–31)^. Likewise, clinical studies have reported reduced cognitive ability in the offspring of mothers with suboptimal folate status^(32,33)^. Over 40 years ago, Gross *et al.* ^(34)^ showed that children born to mothers with diagnosed folate-related megaloblastic anaemia in the third trimester of pregnancy had abnormal neurodevelopment and lower intellectual abilities^(34)^. Several decades later, a longitudinal study of 256 mother–child pairs linked maternal folate deficiency in later pregnancy with reduced brain volume in the children aged 6–8 years, as measured using MRI^(35)^. However, the evidence is inconsistent, as other observational studies have found no significant associations of folate status in pregnancy with cognitive performance^(36)^ or infant neurodevelopment^(37)^.

The very few randomised trials conducted to date in this area have investigated the effect of multiple micronutrient supplements, not folate supplementation alone^(13)^. The FASSTT Offspring trial studied the effect of folic acid supplementation during the 2nd and 3rd trimesters on the subsequent cognitive performance of the child using validated assessment tools^(38)^. The children of folic acid-treated mothers scored significantly higher than the placebo group in several cognitive domains at 3 and 7 years. When compared with nationally representative samples of British children at 7 years, test scores were significantly higher in children from folic acid-treated mothers for Verbal IQ, Performance IQ, General Language and Full-Scale IQ^(38)^, suggesting a role for folate-mediated epigenetic changes in genes related to brain development^(27,28)^.

Whilst folate-mediated epigenetic changes in genes related to brain development and function offer a biological basis to link maternal folate with offspring cognitive effects, this area of research is still in its infancy. Further carefully designed randomised trials with validated cognitive tests and ideally incorporating objective brain imaging techniques are warranted. Such studies could shed further light on the links between folate nutrition during pregnancy and offspring health outcomes and the underpinning epigenetic mechanisms. Personalised approaches, such as accurate dietary assessment of folate intake, or minimally invasive testing for folate status, are needed to identify individuals at risk of folate insufficiency or deficiency. More research is required in the case of 10 % of the worldwide population who are homozygous for the methylenetetrahydrofolate reductase C677T polymorphism as they have impaired folate metabolism and may thus have higher dietary requirements for folate^(39)^.

## Genes affecting vitamin B_12_ metabolism and potential role in cardiometabolic disease

Cardiometabolic diseases such as obesity, type 2 diabetes and CVD are worldwide health problems and are now increasingly prevalent^(40)^. The role of vitamins in cardiometabolic disease risk has more recently been the subject of research in this area. While most of the research has concentrated on vitamin D^(41)^, interest in vitamin B_12_ as a modulator of metabolic disease risk has been increasing^(42)^.

### Vitamin B_12_ deficiency and cardiometabolic diseases

The relationship between low plasma or serum vitamin B_12_ concentrations and cardiometabolic phenotypes could be the result of several mechanisms^(43)^. Vitamin B_12_ is a co-enzyme which converts methylmalonyl-CoA to succinyl-CoA, a critical step in the metabolism of odd-chain fatty acids. If this reaction cannot occur, methylmalonyl-CoA levels elevate, inhibiting the rate-limiting enzyme of fatty acid oxidation (carnitine palmitoyltransferase), leading to lipogenesis and insulin resistance^(44)^. On the other hand, reduced vitamin B_12_ concentrations in obese individuals could result from a nutrient-poor diet and increased nutrient requirements related to increased body size^(45,46)^. Additionally, deficiency of vitamin B_12_ can impair the remethylation of homocysteine in the methionine cycle resulting in raised homocysteine levels^(47)^, which is associated with an increased risk of CVD^(48)^.

Although vitamin B_12_ deficiency is associated with a wide range of chronic diseases and conditions, the relationship between low vitamin B_12_ status and cardiometabolic-related traits has remained inconsistent in numerous studies^(42,49)^. Polymorphisms in the key genes coding for proteins involved in the absorption, cellular uptake and intracellular metabolism of vitamin B_12_ may emerge as a feasible explanation for the vitamin B_12_ variability observed within these studies^(50)^. Genetic studies have identified several genetic variants related to vitamin B_12_ status, during the last few years^(43)^. At present, only three studies in European populations have investigated the effect of genetically instrumented vitamin B_12_ concentrations on cardiometabolic traits such as BMI^(51)^, blood pressure^(52)^ and cardiometabolic risk^(53)^, indicating the need to study more diverse ethnic groups.

Vitamin B_12_ concentrations, which vary widely among individuals, are responsive to changes in diet and are dependent on the quality and consumption of animal protein^(54)^. Therefore, controlling diet is recommended in preventing vitamin B_12_ deficiency^(55)^. As mentioned previously, deficiencies of vitamin B_12_ and folate during pregnancy can affect DNA methylation^(56)^, suggesting that interactions between genes and nutrients may also play a role in the development of cardiometabolic disease. Given that the genetic make-up varies from individual to individual, and there is variation in genetic heritage and food consumed worldwide, it is vital to examine the interactive effects between dietary factors and genetics on vitamin B_12_ concentrations and metabolic traits (nutrigenetics)^(57)^.

### Gene–nutrient interactions with vitamin B_12_

Whilst only a few nutrigenetics studies have been carried out in lower-middle-income countries, a large-scale collaborative project called the ‘gene–nutrient interactions (GeNuIne) Collaboration’ was established^(57,58)^. One of the objectives of the overall project was to investigate the effect of gene–nutrient interactions on vitamin B_12_ concentrations and cardiometabolic traits using population-based studies from various ethnic groups. The first vitamin B_12_ pilot study of the GeNuIne Collaboration was the Genetics of Obesity and Diabetes study. This study explored the relationship of vitamin B_12_ status and metabolic traits in 109 healthy Sinhalese adults in Colombo, Sri Lanka^(59)^. Genetic risk scores (GRS) were derived using ten vitamin B_12_-associated SNP (B12-GRS). While there was a significant association between the B12-GRS and B12 concentrations, there was no significant impact of genetically instrumented B12 concentrations on any of the metabolic traits. However, there was a significant interaction between the B12-GRS and protein energy (%) on waist circumference, suggesting that a genetically lowered vitamin B_12_ concentration may have an impact on central obesity in the presence of lower dietary protein intakes.

The Minangkabau Indonesia Study is another nutrigenetic study in South East Asians conducted in Indonesia as part of the GeNuIne Collaboration^(60)^. The B12-GRS was constructed based on nine vitamin B_12_ SNP, and a metabolic disease-related GRS (metabolic-GRS) was developed based on nine metabolic disease-related SNP. The study examined the relationship of these risk scores with vitamin B_12_ levels and different metabolic traits in 117 healthy Minangkabau Indonesian women. This study demonstrated the impact of genetically instrumented B_12_ concentrations on HbA1C levels, a marker of glycaemic control^(61)^, through the influence of dietary fibre intake.

Given the increased prevalence of type 2 diabetes among Asian Indians, the Chennai Urban Rural Epidemiology Study from South India examined the association between two commonly studied fat mass and obesity-associated gene (*FTO*) SNP on metabolic traits and vitamin B_12_ concentrations in 548 Asian Indians (GeNuIne Collaboration). The study identified significant associations between the two *FTO* SNP not only with a higher risk of obesity but also with a lower vitamin B_12_ concentration^(62)^. These results suggest that increases in BMI could potentially contribute to the adverse health effects associated with vitamin B_12_ deficiency. A more recent study has shown that long-term supplementation with vitamin B_12_ influences the regulation of several type 2 diabetes-associated genes by methylation of miRNA-coding gene, miR21, and could thus epigenetically regulate the risk of obesity, insulin resistance and type 2 diabetes^(63)^.

While these findings are fascinating and highlight novel possibilities of gene–nutrient interactions, they need to be replicated in an independent cohort utilising a larger number of samples. Further understanding of the role of these gene–diet interactions at the molecular level is necessary before diets can be personalised according to varying ethnicity or genotype.

## Importance of microbial enterotypes in personalised obesity management

As the prevalence of obesity has reached epidemic proportions globally, the search for effective management continues. Through multiple dietary intervention studies to reduce energy content, it has become evident that there is considerable weight loss variation among participants^(64,65)^, indicating that no ‘one diet fits all’. Accordingly, dietary weight loss success is likely dependent on several individual characteristics, including host genetics and gut microbiota^(66)^. While gut microbiota-modulating interventions can alleviate obesity in mice^(67)^, similar causal findings appear absent in human clinical trials. However, this has also revealed the enormous variability of the human gut microbiome within and across populations.

To reduce complexity, researchers have proposed stratification of individuals according to distinct gut microbiota composition types termed ‘enterotypes’^(68)^. Primarily two enterotypes have consistently been found across populations; one type dominated by *Prevotella* species, the other by *Bacteroides* species^(69)^. The establishment of these types seems to occur during early childhood and to be highly influenced by long-term dietary habits^(70)^, with the *Prevotella* enterotype associated with a carbohydrate- and fibre-rich diet. Conversely, the *Bacteroides* enterotype is associated with a ‘Western diet’ low in fibre, and high in fats and refined sugars^(71)^. Factors such as age, sex, cultural background and geography have been found to have little influence on the establishment of enterotypes^(69)^.

### Enterotype predicts success in weight loss when consuming high-fibre diets

Following the discovery of enterotypes, epidemiological studies have found conflicting associations between enterotypes and metabolic health^(72,73)^. As the enterotypes seem to differ in their ability to degrade dietary fibre^(69)^, it is adjacent to speculate that metabolic responses of the enterotypes depend on dietary compositions^(74)^. Recent dietary intervention studies with healthy European subjects point to a more beneficial role of a high-fibre diet for individuals with a *Prevotella* enterotype than individuals with the *Bacteroides* enterotype.

In 2015, Kovatcheva and co-workers convincingly linked enterotypes to glucose metabolism after consuming a high-fibre, barley-rich diet for 3 d^(75)^. Specifically, they found that individuals with the *Prevotella* enterotype improved their enzymatic capacity for fibre degradation and glucose metabolism as a result of the diet, an effect not seen among *Bacteroides* enterotype subjects.

Since then, Hjorth and co-workers reanalysed a 26-week *ad libitum* study with a fibre-rich New Nordic Diet (NND) and an average Danish diet, lower in fibre (43·3 *v*. 28·6 g/10 MJ), and predicted dietary success based on pre-treatment faecal *Prevotella*:*Bacteroides* (P:B) ratio^(76)^. While the high P:B group lost weight on NND compared with the control diet, the low P:B group did not. Interestingly, during the 1-year follow-up period, subjects with the high P:B ratio who changed from the control diet to the recommended NND managed to maintain their weight loss, whereas subjects with the low P:B ratio who changed from the control diet to the NND regained weight.

To validate these findings, two other dietary intervention studies aimed at weight loss were stratified by enterotype^(77,78)^. Similarly, these studies observed that subjects who consumed a high-fibre diet for 6 months lost more weight if they had a high P:B ratio *v*. a low P:B ratio. Furthermore, in a 6-week *ad libitum* study comparing whole grains with refined wheat (fibre intake: 33 g/d *v*. 23 g/d), a 1·8 kg weight loss was observed among participants with high *Prevotella* abundance, and no difference in the low *Prevotella* group^(79)^. The finding suggests that specific whole-grain fibres, such as arabinoxylans, benefit the *Prevotella* enterotype, likely due to a beneficial match between species enzymatic degradation capacity of especially the prevalent *Prevotella* species, *P. copri* ^(80)^. In comparison, the colonic functional and ecological properties seem less uniform among subjects with a *Bacteroides* enterotype^(81)^.

### Enterotypes at lower taxonomical levels and host digestive capacity

Until recently, the majority of dietary intervention studies have been limited to genus level taxonomy by 16S rRNA gene sequencing. Although with continuous advances in the field of sequencing, species-level taxonomy is now widespread, revealing a large inter-individual variation in *Bacteroides* and *Prevotella* species^(74)^. Moreover, at the strain level, recent pioneering microbiome studies demonstrated that *P. copri* is not a monotypic species, but encompasses four distinct clades with substantial functional diversity differences including the ability to degrade whole-grain fibres^(82)^. Consequently, if weight loss is affected by the colonic bacterial enzymatic capacity, future enterotype studies need deep-level sequencing to explain potential weight loss variability further.

Another intriguing factor in obesity management is the ability of the host to degrade the major polysaccharide component of diets, starch^(83)^. If dietary starches escape host digestion in the small intestine, it will primarily feed *Prevotella* and *Bacteroides* species located distally in the gut. Amylase secreted from the salivary glands and pancreas determines the amount of starch escaping host digestion, and genetically the salivary amylase gene (AMY-1) exhibits some of the greatest copy numbers (CN) of any human gene^(84)^. Stratification of participants of the 26-week NND study (discussed above) according to AMY-1 CN (i.e. low and high AMY-1 CN groups, respectively) further explained weight loss variability^(85)^. Here, the majority of weight loss difference between enterotypes was observed in the low AMY-1 CN group with a strong linear relationship between P:B ratio and weight loss, which was not found for the high AMY-1 CN group. This indicates that not only dietary fibre but also the total pool of fermentable substrate reaching the colon play a role in the weight loss difference between the enterotypes.

Moving this field forward, there is furthermore a need to understand the underlying mechanisms linking microbial fibre fermentation to host metabolic alterations. To elucidate this, microbial metabolites produced in the colon that enter circulation and interact with host cells need to be investigated (e.g. SCFA)^(86)^. In conclusion, microbial enterotypes are currently highly interesting biomarkers for explaining variability in weight loss upon intake of dietary fibre and whole-grain-rich diets^(87)^. Soon, it might be possible to complement personalised dietary weight-loss strategies with microbial enterotype tests, thus ensuring dietary preferences of both the individual and the colonic microbes. Such a personalised approach may, in fact, support dietary adherence and long-term weight maintenance.

## Advanced technologies for food image recognition in nutrient intake assessment

Dietary assessment is a crucial step in the real-world deployment of any personalised nutrition programme. The ability of an individual to track their food intake plays a role in self-monitoring as a critical aspect of behaviour change^(88)^. It can also provide a professional dietitian with information on how a client is adhering to their individualised meal plan. However, assessing dietary intake with traditional methods carries considerable costs and burden to the individual. Such methods are also prone to errors as they often rely on self-reporting^(89)^.

Advanced solutions are needed to objectively quantify food and beverage intake^(90)^. Food image recognition is a promising strategy because most individuals own a smartphone with a camera, so the barrier to entry is low, and it can reach a large population. However, automatically recognising food items from images is a challenging computer vision problem due to a variety of issues: (1) foods are typically deformable objects, (2) foods can lose their visual information during preparation, (3) different foods can appear visually similar, (4) the same food can appear differently depending on the lighting or angle and (5) limited amount of visual information for beverages^(91)^. Only after foods are visually recognised, can they be reliably linked to a food composition database^(92)^.

The introduction of the Pittsburgh Fast-Food Image Dataset in 2009 facilitated early research in this area based on manual recognition methods, but these approaches mostly achieved only 10–40 % classification accuracy^(93,94)^. In 2014, deep learning was first used to recognise food images. Deep learning, or deep neural networks, allows computational models composed of multiple processing layers to learn relevant image features through training on a set of input images^(95,96)^. Deep convolutional neural networks are inspired by the visual system of animals, where individual neurons assess the visual input by reacting to overlapping regions in the visual field^(97)^. Because they can classify each pixel of the image, they can recognise any number of items, along with their location and size, allowing for food volume and food weight estimation. This approach has achieved substantially better results than other methods, resulting in an increased focus on deep learning in recent research^(98,99)^.

A novel deep learning architecture for food image recognition, called NutriNet, has been developed by Mezgec and Koroušić Seljak^(91)^. It is a modification of the well-known AlexNet architecture^(100)^, with increased image size and an additional convolutional layer at the beginning of the neural network^(91)^. NutriNet was first trained on 225 953 freely available images that were downloaded from the Internet and organised into appropriate food classes (520 unique food items). When tested against three popular deep learning architectures of the time (AlexNet^(100)^, GoogLeNet^(101)^ and ResNet^(102)^), NutriNet was found to be superior to AlexNet and GoogLeNet and faster to train than all three of the other architectures. The deep learning approach was then used to recognise any number of items in a single food image using a training set obtained from the ‘fake food buffet’^(103)^, which is visually similar to real food. Fully convolutional networks, introduced by Long *et al*.^(104)^, were applied to perform semantic segmentation, partitioning the image into logical parts and classifying each part on a pixel level. Due to the complexity of food images, an fully convolutional network variant that can segment images at the finest grain (fully convolutional network-8s)^(104)^ was used to train a model on the fake food buffet image data set. Output predictions of the trained model were compared with the ground-truth labels using the pixel accuracy measure^(104)^, and the final accuracy of the trained fully convolutional network-8s model was 92·18 %.

In recent years, deep learning has been validated numerous times as a suitable solution for recognising food images^(98)^. Availability of food image data sets has been improving^(105–107)^, although there is a need for validation against data sets from different regions across the world. Future work will focus on real-world food images, which exhibit more variance compared with the test images used in the research environment. In the future, such technology could be used to improve dietary assessment in clinical trials. For example, it can play a role in human studies in the areas mentioned above, where accurate quantification of folate, vitamin B_12_ and energetic intake is critical. Consumer wellness apps can also apply this solution in the future, improving the dietary assessment of individuals and facilitating self-monitoring towards positive behaviour change.

## Looking towards the future: considerations to facilitate individualised nutrition guidance

From the four examples discussed here, it is clear that scientific evidence about individualised responses to nutrient intakes continues to emerge. By recognising these sources of individual variation, we have the potential to improve the conduct and interpretation of clinical trials. For example, in studies of folate status and epigenetics, dietary intake assessment may be enhanced by the use of an advanced technique such as the one described here. In obesity research, understanding genes related to salivary amylase and vitamin B_12_ metabolism, and gut microbiota composition, may enable better prediction of responders and non-responders and dietary adherence to an intervention could be assessed by photo recognition.

Such approaches can also play a role in the development of personalised nutrition programmes in the public health arena. For maternal health, such an app could help to identify women at risk of folate deficiency, providing them with guidance on how to identify foods with adequate folate, or supplementation options that meet their individual preferences. A weight management app could integrate automated dietary intake assessment with information on genotype and microbiome composition to provide a user with more pertinent and individualised guidance.

As new information comes to light, such as that described in the four examples in this paper, confidence in individualised nutrition guidance is likely to increase. In the meantime, companies and organisations developing personalised nutrition programmes must take science-based, ethical and rigorous approaches in developing their guidance^(3,108)^. In this way, personalised nutrition approaches can maintain their credibility, provide maximum benefit to the individual and advance public health.

## References

[1] Kanter M & Desrosiers A (2019) Personalized wellness past and future: Will the science and technology coevolve?. Nutr Today 54, 174–181.

[2] Pavlidis C , Lanara Z , Balasopoulou A , et al. (2015) Meta-analysis of genes in commercially available nutrigenomic tests denotes lack of association with dietary intake, nutrient-related pathologies. OMICS 19, 512–520. 26348710 10.1089/omi.2015.0109

[3] Adams SH , Anthony JC , Carvajal R , et al. (2020) Perspective: guiding principles for the implementation of personalized nutrition approaches that benefit health and function. Adv Nutr 11, 25–34. 31504115 10.1093/advances/nmz086 PMC7442375

[4] Rozga M , Latulippe ME & Steiber A (2020) Advancements in personalized nutrition technologies: guiding principles for registered dietitian nutritionists. J Acad Nutr Diet 120, 1074–1085. 32299678 10.1016/j.jand.2020.01.020

[5] Patnala R , Clements J & Batra J (2013) Candidate gene association studies: a comprehensive guide to useful *in silico* tools. BMC Genet 14, 39. 23656885 10.1186/1471-2156-14-39 PMC3655892

[6] Witte JS (2010) Genome-wide association studies and beyond. Annu Rev Public Health 31, 9–20. 20235850 10.1146/annurev.publhealth.012809.103723 PMC3997166

[7] Frazier-Wood AC (2015) Dietary patterns, genes, and health: challenges and obstacles to be overcome. Curr Nutr Rep 4, 82–87. 25664222 10.1007/s13668-014-0110-6 PMC4315873

[8] Ordovas JM , Ferguson LR , Tai ES , et al. (2018) Personalised nutrition and health. BMJ 361, bmj.k2173. 29898881 10.1136/bmj.k2173 PMC6081996

[9] McDonald C & Thorne-Lyman A (2017) The importance of the first 1,000 days: an epidemiological perspective. In The Biology of the First 1000 days, pp. 3–16 [ Karakochuk C , Whitfield K , Green T , et al., editors]. Florida: CRC Press.

[10] Bailey LB , Stover PJ , McNulty H , et al. (2015) Biomarkers of nutrition for development—folate review. J Nutr 147, 1636S–1680S. 10.3945/jn.114.206599 PMC4478945 26451605

[11] MRC Vitamin Study Research Group (1991) Prevention of neural tube defects: results of the Medical Research Council Vitamin Study. Lancet 338, 131–137. 1677062

[12] Czeizel AE & Dudás I (1992) Prevention of the first occurrence of neural-tube defects by periconceptional vitamin supplementation. New Engl J Med 327, 1832–1835. 1307234 10.1056/NEJM199212243272602

[13] Caffrey A , McNulty H , Irwin RE , et al. (2019) Maternal folate nutrition and offspring health: evidence and current controversies. Proc Nutr Soc 78, 208–220. 30585558 10.1017/S0029665118002689

[14] Roffman JL (2018) Neuroprotective effects of prenatal folic acid supplementation: why timing matters. JAMA Psychiat 75, 747–748. 10.1001/jamapsychiatry.2018.0378 PMC6777839 29801055

[15] Gilmore JH , Shi F , Woolson SL , et al. (2012) Longitudinal development of cortical and subcortical gray matter from birth to 2 years. Cereb Cortex 22, 2478–2485. 22109543 10.1093/cercor/bhr327 PMC3464410

[16] Irwin RE , Pentieva K , Cassidy T , et al. (2016) The interplay between DNA methylation, folate and neurocognitive development. Epigenomics 8, 863–879. 27319574 10.2217/epi-2016-0003

[17] Georgieff MK , Brunette KE & Tran PV (2015) Early life nutrition and neural plasticity. Dev Psychopathol 27, 411–423. 25997762 10.1017/S0954579415000061 PMC4443711

[18] Gabbianelli R & Damiani E (2018) Epigenetics and neurodegeneration: role of early-life nutrition. J Nutr Biochem 57, 1–13. 29529489 10.1016/j.jnutbio.2018.01.014

[19] Kok DE , Steegenga WT & Mckay JA (2018) Folate and epigenetics: why we should not forget bacterial biosynthesis. Epigenomics 10, 1147–1150. 30238776 10.2217/epi-2018-0117

[20] Armstrong L (2014) Epigenetics. New York: Garland Science.

[21] Numata S , Ye T , Hyde TM , et al. (2012) DNA methylation signatures in development and aging of the human prefrontal cortex. Am J Hum Genet 90, 260–272. 22305529 10.1016/j.ajhg.2011.12.020 PMC3276664

[22] Joubert BR , den Dekker HT , Felix JF , et al. (2016) Maternal plasma folate impacts differential DNA methylation in an epigenome-wide meta-analysis of newborns. Nat Commun 7, 10577. 26861414 10.1038/ncomms10577 PMC4749955

[23] Haggarty P , Hoad G , Campbell DM , et al. (2013) Folate in pregnancy and imprinted gene and repeat element methylation in the offspring. Am J Clin Nutr 97, 94–99. 23151531 10.3945/ajcn.112.042572

[24] Steegers-Theunissen RP , Obermann-Borst SA , Kremer D , et al. (2009) Periconceptional maternal folic acid use of 400 μg per day is related to increased methylation of the IGF2 gene in the very young child. PLoS One 4, e7845. 19924280 10.1371/journal.pone.0007845 PMC2773848

[25] James P , Sajjadi S , Tomar AS , et al. (2018) Candidate genes linking maternal nutrient exposure to offspring health via DNA methylation: A review of existing evidence in humans with specific focus on one-carbon metabolism. Int J Epidemiol 47, 1910–1937. 30137462 10.1093/ije/dyy153 PMC6280938

[26] McNulty B , McNulty H , Marshall B , et al. (2013) Impact of continuing folic acid after the first trimester of pregnancy: findings of a randomized trial of folic acid supplementation in the second and third trimesters. Am J Clin Nutr 98, 92–98. 23719554 10.3945/ajcn.112.057489

[27] Caffrey A , Irwin RE , McNulty H , et al. (2018) Gene-specific DNA methylation in newborns in response to folic acid supplementation during the second, third trimesters of pregnancy: epigenetic analysis from a randomized controlled trial. Am J Clin Nutr 107, 566–575. 29635492 10.1093/ajcn/nqx069

[28] Irwin RE , Thursby S-J , Ondičová M , et al. (2019) A randomized controlled trial of folic acid intervention in pregnancy highlights a putative methylation-regulated control element at ZFP57. Clin Epigenet 11, 31. 10.1186/s13148-019-0618-0 PMC6380035 30777123

[29] Julvez J , Fortuny J , Mendez M , et al. (2009) Maternal use of folic acid supplements during pregnancy and four-year-old neurodevelopment in a population-based birth cohort. Paediatr Perinat Epidemiol 23, 199–206. 19775381 10.1111/j.1365-3016.2009.01032.x

[30] Roth C , Magnus P , Schjølberg S , et al. (2011) Folic acid supplements in pregnancy and severe language delay in children. JAMA 306, 1566–1573. 21990300 10.1001/jama.2011.1433 PMC3780384

[31] Villamor E , Rifas-Shiman SL , Gillman MW , et al. (2012) Maternal intake of methyl-donor nutrients and child cognition at 3 years of age. Paediatr Perinat Epidemiol 26, 328–335. 22686384 10.1111/j.1365-3016.2012.01264.x PMC3375854

[32] Schlotz W , Jones A , Phillips DIW , et al. (2010) Lower maternal folate status in early pregnancy is associated with childhood hyperactivity and peer problems in offspring. J Child Psychol Psych 51, 594–602. 10.1111/j.1469-7610.2009.02182.x PMC2862762 19874428

[33] Murphy MM , Fernandez-Ballart JD , Molloy AM , et al. (2016) Moderately elevated maternal homocysteine at preconception is inversely associated with cognitive performance in children 4 months and 6 years after birth. Matern Child Nutr 13, e12289. 10.1111/mcn.12289 PMC6866183 26817572

[34] Gross RL , Newberne PM & Reid JVO (1974) Adverse effects on infant development associated with maternal folic acid deficiency. Nutr Rep Int 10, 241–248.

[35] Ars CL , Nijs IM , Marroun HE , et al. (2016) Prenatal folate, homocysteine and vitamin B12 levels and child brain volumes, cognitive development and psychological functioning: the Generation R Study. Brit J Nutr 122, S1–S9. 31638501 10.1017/S0007114515002081

[36] Tamura T , Goldenberg RL , Chapman VR , et al. (2005) Folate status of mothers during pregnancy and mental and psychomotor development of their children at five years of age. Pediatrics 116, 703–708. 16140711 10.1542/peds.2004-2189

[37] Wu BTF , Dyer RA , King DJ , et al. (2012) Early second trimester maternal plasma choline and betaine are related to measures of early cognitive development in term infants. PLoS One 7, e43448. 22916264 10.1371/journal.pone.0043448 PMC3423345

[38] McNulty H , Rollins M , Cassidy T , et al. (2019) Effect of continued folic acid supplementation beyond the first trimester of pregnancy on cognitive performance in the child: a follow-up study from a randomized controlled trial (FASSTT Offspring Trial). BMC Med 17, 196. 31672132 10.1186/s12916-019-1432-4 PMC6823954

[39] Shelnutt KP , Kauwell GPA , Chapman CM , et al. (2003) Folate status response to controlled folate intake is affected by the methylenetetrahydrofolate reductase 677C-->T polymorphism in young women. J Nutr 133, 4107–4111. 14652356 10.1093/jn/133.12.4107

[40] Virani SS , Alonso A , Benjamin EJ , et al. (2020) Heart disease and stroke statistics—2020 update: a report from the american heart association. Circulation 141, e139–e596. 31992061 10.1161/CIR.0000000000000757

[41] Gouni-Berthold I & Berthold HK (2020) Vitamin D and vascular disease. Curr Vasc Pharmacol 19, 250–268. 10.2174/1570161118666200317151955 32183681

[42] Wiebe N , Field CJ & Tonelli M (2018) A systematic review of the vitamin B12, folate and homocysteine triad across body mass index. Obes Rev 19, 1608–1618. 30074676 10.1111/obr.12724

[43] Surendran S , Adaikalakoteswari A , Saravanan P , et al. (2018) An update on vitamin B12-related gene polymorphisms and B12 status. Genes Nutr 13, 2. 29445423 10.1186/s12263-018-0591-9 PMC5801754

[44] Rush EC , Katre P & Yajnik CS (2014) Vitamin B12: one carbon metabolism, fetal growth and programming for chronic disease. Eur J Clin Nutr 68, 2–7. 24219896 10.1038/ejcn.2013.232

[45] Pinhas-Hamiel O , Doron-Panush N , Reichman B , et al. (2006) Obese children and adolescents: A risk group for low vitamin B12 concentration. Arch Pediatr Adolesc Med 160, 933–936. 16953016 10.1001/archpedi.160.9.933

[46] MacFarlane AJ , Greene-Finestone LS & Shi Y (2011) Vitamin B-12 and homocysteine status in a folate-replete population: results from the Canadian Health Measures Survey. Am J Clin Nutr 94, 1079–1087. 21900461 10.3945/ajcn.111.020230

[47] Selhub J (1999) Homocysteine metabolism. Ann Rev Nutr 19, 217–246. 10448523 10.1146/annurev.nutr.19.1.217

[48] Wald DS , Law M & Morris JK (2002) Homocysteine and cardiovascular disease: evidence on causality from a meta-analysis. BMJ 325, 1202. 12446535 10.1136/bmj.325.7374.1202 PMC135491

[49] Rafnsson SB , Saravanan P , Bhopal RS , et al. (2011) Is a low blood level of vitamin B12 a cardiovascular and diabetes risk factor? a systematic review of cohort studies. Eur J Nutr 50, 97–106. 20585951 10.1007/s00394-010-0119-6

[50] Quadros EV (2010) Advances in the understanding of cobalamin assimilation and metabolism. Br J Haematol 148, 195–204. 19832808 10.1111/j.1365-2141.2009.07937.x PMC2809139

[51] Allin KH , Friedrich N , Pietzner M , et al. (2017) Genetic determinants of serum vitamin B12 and their relation to body mass index. Eur J Epidemiol 32, 125–134. 27995393 10.1007/s10654-016-0215-x PMC5374184

[52] Husemoen LLN , Skaaby T , Thuesen BH , et al. (2016) Mendelian randomisation study of the associations of vitamin B12 and folate genetic risk scores with blood pressure and fasting serum lipid levels in three Danish population-based studies. Eur J Clin Nutr 70, 613–619. 26908422 10.1038/ejcn.2016.5

[53] Moen G-H , Qvigstad E , Birkeland KI , et al. (2018) Are serum concentrations of vitamin B-12 causally related to cardiometabolic risk factors and disease? a mendelian randomization study. Am J Clin Nutr 108, 398–404. 29982347 10.1093/ajcn/nqy101

[54] Watanabe F , Yabuta Y , Tanioka Y , et al. (2013) Biologically active vitamin B12 compounds in foods for preventing deficiency among vegetarians and elderly subjects. J Agric Food Chem 61, 6769–6775. 23782218 10.1021/jf401545z

[55] Watanabe F (2007) Vitamin B12 sources and bioavailability. Exp Biol Med 232, 1266–1274. 10.3181/0703-MR-67 17959839

[56] Yajnik CS & Deshmukh US (2012) Fetal programming: Maternal nutrition and role of one-carbon metabolism. Rev Endocr Metab Disord 13, 121–127. 22415298 10.1007/s11154-012-9214-8

[57] Vimaleswaran KS (2020) A nutrigenetics approach to study the impact of genetic and lifestyle factors on cardiometabolic traits in various ethnic groups: Findings from the GeNuIne Collaboration. Proc Nutr Soc 79, 194–204. 32000867 10.1017/S0029665119001186

[58] Vimaleswaran KS (2017) Gene–nutrient interactions on metabolic diseases: findings from the GeNuIne Collaboration. Nutr Bull 42, 80–86.

[59] Surendran S , Alsulami S , Lankeshwara R , et al. (2020) A genetic approach to examine the relationship between vitamin B12 status and metabolic traits in a South Asian population. Int J Diabetes Dev Ctries 40, 21–31.

[60] Surendran S , Aji AS , Ariyasra U , et al. (2019) A nutrigenetic approach for investigating the relationship between vitamin B12 status and metabolic traits in Indonesian women. J Diabetes Metab Disord 18, 389–399. 31890664 10.1007/s40200-019-00424-z PMC6914754

[61] Borg R , Persson F , Siersma V , et al. (2018) Interpretation of HbA1c in primary care and potential influence of anaemia and chronic kidney disease: an analysis from the Copenhagen Primary Care Laboratory (CopLab) Database. Diabet Med 35, 1700–1706. 29985535 10.1111/dme.13776

[62] Surendran S , Jayashri R , Drysdale L , et al. (2019) Evidence for the association between FTO gene variants and vitamin B12 concentrations in an Asian Indian population. Genes Nutr 14, 26. 31516636 10.1186/s12263-019-0649-3 PMC6728975

[63] Yadav DK , Shrestha S , Lillycrop KA , et al. (2018) Vitamin B12 supplementation influences methylation of genes associated with Type 2 diabetes and its intermediate traits. Epigenomics 10, 71–90. 29135286 10.2217/epi-2017-0102

[64] Poulsen SK , Due A , Jordy AB , et al. (2014) Health effect of the New Nordic Diet in adults with increased waist circumference: A 6-mo randomized controlled trial. Am J Clin Nutr 99, 35–45. 24257725 10.3945/ajcn.113.069393

[65] Hess AL , Benítez-Páez A , Blædel T , et al. (2019) The effect of inulin and resistant maltodextrin on weight loss during energy restriction: A randomised, placebo-controlled, double-blinded intervention. Eur J Nutr 59, 2507–2524. 31605197 10.1007/s00394-019-02099-x

[66] Zeevi D , Korem T , Zmora N , et al. (2015) Personalized trition by prediction of glycemic responses. Cell 163, 1079–1094. 26590418 10.1016/j.cell.2015.11.001

[67] Sonnenburg JL & Bäckhed F (2016) Diet–microbiota interactions as moderators of human metabolism. Nature 535, 56–64. 27383980 10.1038/nature18846 PMC5991619

[68] Arumugam M , Raes J , Pelletier E , et al. (2011) Enterotypes of the human gut microbiome. Nature 473, 174–180. 21508958 10.1038/nature09944 PMC3728647

[69] Costea PI , Hildebrand F , Arumugam M , et al. (2018) Enterotypes in the landscape of gut microbial community composition. Nat Microbiol 3, 8–16. 29255284 10.1038/s41564-017-0072-8 PMC5832044

[70] Wu GD , Chen J , Hoffmann C , et al. (2011) Linking long-term dietary patterns with gut microbial enterotypes. Science 334, 105–108. 21885731 10.1126/science.1208344 PMC3368382

[71] Vangay P , Johnson AJ , Ward TL , et al. (2018) US immigration westernizes the human gut microbiome. Cell 175, 962–972.e10. 30388453 10.1016/j.cell.2018.10.029 PMC6498444

[72] Pedersen HK , Gudmundsdottir V , Nielsen HB , et al. (2016) Human gut microbes impact host serum metabolome and insulin sensitivity. Nature 535, 376–381. 27409811 10.1038/nature18646

[73] Ley RE (2016) Prevotella in the gut: choose carefully. Nat Rev Gastro Hepat 13, 69–70. 10.1038/nrgastro.2016.4 26828918

[74] Christensen L , Roager HM , Astrup A , et al. (2018) Microbial enterotypes in personalized nutrition and obesity management. Am J Clin Nutr 108, 645–651. 30239555 10.1093/ajcn/nqy175

[75] Kovatcheva-Datchary P , Nilsson A , Akrami R , et al. (2015) Dietary fiber-induced improvement in glucose metabolism is associated with increased abundance of *Prevotella* . Cell Metab 22, 971–982. 26552345 10.1016/j.cmet.2015.10.001

[76] Hjorth MF , Roager HM , Larsen TM , et al. (2018) Pre-treatment microbial Prevotella -to- Bacteroides ratio, determines body fat loss success during a 6-month randomized controlled diet intervention. Int J Obes 42, 580–583. 10.1038/ijo.2017.220 PMC5880576 28883543

[77] Hjorth MF , Blædel T , Bendtsen LQ , et al. (2019) *Prevotella* -to- *Bacteroides* ratio predicts body weight and fat loss success on 24-week diets varying in macronutrient composition and dietary fiber: results from a post-hoc analysis. Int J Obes 43, 149–157. 10.1038/s41366-018-0093-2 PMC6331389 29777234

[78] Hjorth MF , Christensen L , Kjølbæk L , et al. (2020) Pretreatment *Prevotella* -to- *Bacteroides* ratio and markers of glucose metabolism as prognostic markers for dietary weight loss maintenance. Eur J Clin Nutr 74, 338–347. 31285554 10.1038/s41430-019-0466-1

[79] Christensen L , Vuholm S , Roager HM , et al. (2019) *Prevotella* abundance predicts weight loss success in healthy, overweight adults consuming a whole-grain diet *ad libitum*: a *post hoc* analysis of a 6-wk randomized controlled trial. J Nutr 149, 2174–2181. 31504699 10.1093/jn/nxz198

[80] De Filippis F , Pasolli E , Tett A , et al. (2019) Distinct genetic, functional traits of human intestinal Prevotella copri strains are associated with different habitual diets. Cell Host Microbe 25, 444–453.e3. 30799264 10.1016/j.chom.2019.01.004

[81] De Paepe K , Verspreet J , Courtin CM , et al. (2020) Microbial succession during wheat bran fermentation and colonisation by human faecal microbiota as a result of niche diversification. ISME J 14, 584–596. 31712738 10.1038/s41396-019-0550-5 PMC6976558

[82] Tett A , Huang KD , Asnicar F , et al. (2019) The Prevotella copri complex comprises four distinct clades underrepresented in westernized populations. Cell Host Microbe 26, 666–679.e7. 31607556 10.1016/j.chom.2019.08.018 PMC6854460

[83] Falchi M , El-Sayed Moustafa JS , Takousis P , et al. (2014) Low copy number of the salivary amylase gene predisposes to obesity. Nat Genet 46, 492–497. 24686848 10.1038/ng.2939 PMC6485469

[84] Elder PJD , Ramsden DB , Burnett D , et al. (2018) Human amylase gene copy number variation as a determinant of metabolic state. Expert Rev Endocrinol Metab 13, 193–205. 30063422 10.1080/17446651.2018.1499466

[85] Hjorth MF , Christensen L , Larsen TM , et al. (2020) Pretreatment *Prevotella*-to-*Bacteroides* ratio and salivary amylase gene copy number as prognostic markers for dietary weight loss. Am J Clin Nutr 111, 1079–1086. 32034403 10.1093/ajcn/nqaa007

[86] Roager HM & Dragsted LO (2019) Diet-derived microbial metabolites in health and disease. Nutr Bull 44, 216–227.

[87] Ortega-Santos CP & Whisner CM (2019) The key to successful weight loss on a high-fiber diet may be in gut microbiome prevotella abundance. J Nutr 149, 2083–2084. 31584088 10.1093/jn/nxz248

[88] Peterson ND , Middleton KR , Nackers LM , et al. (2014) Dietary self-monitoring and long-term success with weight management. Obesity 22, 1962–1967. 24931055 10.1002/oby.20807 PMC4149603

[89] Burrows TL , Ho YY , Rollo ME , et al. (2019) Validity of dietary assessment methods when compared to the method of doubly labeled water: a systematic review in adults. Front Endocrinol 10, 850. 10.3389/fendo.2019.00850 PMC6928130 31920966

[90] Mezgec S , Eftimov T , Bucher T , et al. (2019) Mixed deep learning and natural language processing method for fake-food image recognition and standardization to help automated dietary assessment. Public Health Nutr 22, 1193–1202. 29623869 10.1017/S1368980018000708 PMC6536832

[91] Mezgec S & Koroušić Seljak B (2017) NutriNet: A deep learning food and drink image recognition system for dietary assessment. Nutrients 9, 657. 10.3390/nu9070657 PMC5537777 28653995

[92] Eftimov T , Korošec P & Koroušić Seljak B (2017) StandFood: standardization of foods using a semi-automatic system for classifying and describing foods according to FoodEx2. Nutrients 9, 542. 10.3390/nu9060542 PMC5490521 28587103

[93] Chen M , Dhingra K , Wu W , et al. (2009) PFID: Pittsburgh fast-food image dataset. In 2009 16th IEEE International Conference on Image Processing (ICIP), pp. 289–292. https://doiorg/10.1109/ICIP.2009.5413511 (accessed May 2020).

[94] Yang S , Chen M , Pomerleau D , et al. (2010) Food recognition using statistics of pairwise local features. In 2010 IEEE Computer Society Conference on Computer Vision, Pattern Recognition, pp. 2249–2256. 10.1109/CVPR.2010.5539907 (accessed May 2020).

[95] LeCun Y , Bengio Y & Hinton G (2015) Deep learning. Nature 521, 436–444. 26017442 10.1038/nature14539

[96] Deng L & Yu D (2014) Deep learning: methods and applications. SIG 7, 197–387.

[97] Hubel DH & Wiesel TN (1962) Receptive fields, binocular interaction and functional architecture in the cat’s visual cortex. J Physiol 160, 106–154.2. 14449617 10.1113/jphysiol.1962.sp006837 PMC1359523

[98] Zhou L , Zhang C , Liu F , et al. (2019) Application of deep learning in food: a review. Comp Rev Food Sci F 18, 1793–1811. 10.1111/1541-4337.12492 33336958

[99] Knez S & Šajn L (2020) Food object recognition using a mobile device: evaluation of currently implemented systems. Trends Food SciTech 99, 460–471.

[100] Krizhevsky A , Sutskever I & Hinton GE (2012) ImageNet classification with deep convolutional neural networks. In Advances in Neural Information Processing Systems 25, pp. 1097–1105 [ Pereira F , Burges CJC , Bottou L , et al., editors]. New York: Curran Associates, Inc.

[101] Szegedy C , Wei Liu , Yangqing Jia , et al. (2015) Going deeper with convolutions. In 2015 IEEE Conference on Computer Vision and Pattern Recognition (CVPR), pp. 1–9. 10.1109/CVPR.2015.7298594 (accessed May 2020).

[102] He K , Zhang X , Ren S , et al. (2016) Deep residual learning for image recognition. In 2016 IEEE Conference on Computer Vision and Pattern Recognition (CVPR), pp. 770–778. 10.1109/CVPR.2016.90 (accessed May 2020).

[103] Bucher T , van der Horst K & Siegrist M (2013) Fruit for dessert. How people compose healthier meals. Appetite 60, 74–80. 23085475 10.1016/j.appet.2012.10.003

[104] Long J , Shelhamer E & Darrell T (2015) Fully convolutional networks for semantic segmentation. In 2015 IEEE Conference on Computer Vision and Pattern Recognition (CVPR), pp. 3431–3440. 10.1109/CVPR.2015.7298965 (accessed May 2020). 27244717

[105] Ciocca G , Napoletano P & Schettini R (2017) Food recognition: A new dataset, experiments, and results. IEEE J Biomed Health 21, 588–598. 10.1109/JBHI.2016.2636441 28114043

[106] Salvador A , Hynes N , Aytar Y , et al. (2017) Learning cross-modal embeddings for cooking recipes and food images. In 2017 IEEE Conference on Computer Vision and Pattern Recognition (CVPR), pp. 3068–3076. 10.1109/CVPR.2017.327 (accessed May 2020).

[107] Cai Q , Li J , Li H , et al. (2019) BTBUFood-60: Dataset for object detection in food field. In 2019 IEEE International Conference on Big Data and Smart Computing (BigComp), pp. 1–4. 10.1109/BIGCOMP.2019.8678916 (accessed May 2020).

[108] Bush CL , Blumberg JB , El-Sohemy A , et al. (2020) Toward the definition of personalized nutrition: A proposal by the american nutrition association. J Am Coll Nutr 39, 5–15. 31855126 10.1080/07315724.2019.1685332

